# Classification of high-grade glioblastoma and single brain metastases using a new SCAT-inception model trained with MRI images

**DOI:** 10.3389/fnins.2024.1349781

**Published:** 2024-03-14

**Authors:** Cheng Lv, Xu-Jun Shu, Hui Chang, Jun Qiu, Shuo Peng, Keping Yu, Sheng-Bo Chen, Hong Rao

**Affiliations:** ^1^School of Mathematics and Computer Sciences, Nanchang University, Nanchang, Jiangxi Province, China; ^2^Department of Neurosurgery, Nanjing Jinling Hospital, Nanjing, Jiangsu Province, China; ^3^Department of Computer and Information Engineering, Henan University, Kaifeng, China; ^4^Department of Critical Care Medicine, The Second People’s Hospital of Yibin, Yibin, Sichuan Province, China; ^5^Department of Computer Science, Jinggangshan University, Ji’an, China; ^6^School of Science and Engineering, Hosei University, Tokyo, Japan

**Keywords:** deep learning, glioblastoma, brain metastasis, MRI, SCAT-inception

## Abstract

**Background and objectives:**

Glioblastoma (GBM) and brain metastasis (MET) are the two most common intracranial tumors. However, the different pathogenesis of the two tumors leads to completely different treatment options. In terms of magnetic resonance imaging (MRI), GBM and MET are extremely similar, which makes differentiation by imaging extremely challenging. Therefore, this study explores an improved deep learning algorithm to assist in the differentiation of GBM and MET.

**Materials and methods:**

For this study, axial contrast-enhanced T1 weight (ceT1W) MRI images from 321 cases of high-grade gliomas and solitary brain metastasis were collected. Among these, 251 out of 270 cases were selected for the experimental dataset (127 glioblastomas and 124 metastases), 207 cases were chosen as the training dataset, and 44 cases as the testing dataset. We designed a new deep learning algorithm called SCAT-inception (Spatial Convolutional Attention inception) and used five-fold cross-validation to verify the results.

**Results:**

By employing the newly designed SCAT-inception model to predict glioblastomas and brain metastasis, the prediction accuracy reached 92.3%, and the sensitivity and specificity reached 93.5 and 91.1%, respectively. On the external testing dataset, our model achieved an accuracy of 91.5%, which surpasses other model performances such as VGG, UNet, and GoogLeNet.

**Conclusion:**

This study demonstrated that the SCAT-inception architecture could extract more subtle features from ceT1W images, provide state-of-the-art performance in the differentiation of GBM and MET, and surpass most existing approaches.

## 1 Introduction

Glioblastoma (GBM) and brain metastasis (MET) are the two most common types of intracranial tumors, posing a significant threat to human health. The incidence rate of GBM is reported to be 3.2–3.5 per 100,000 individuals, whereas MET has a higher incidence rate of 10 per 100,000 individuals (Zhou et al., 2023). GBM constitutes a primary tumor originating from glial cells in the brain and represents the most prevalent brain tumor type. In contrast, MET denotes a secondary tumor resulting from metastasis of malignant cells from other organs to the brain via the bloodstream or lymphatic system. Owing to divergent pathogenic mechanisms, treatment strategies also differ between these two tumor types. Currently, accurate differentiation of GBM and MET relies on pathological examination of tissue specimens (Bae et al., 2020). However, this invasive approach increases surgical risks for patients (Qian et al., 2019).

MRI has been routinely used in brain tumor detection and diagnosis. Contrast-enhanced T1 weighted (ceT1W) MR images can make intracranial lesions bright and provide more details by IV injection of gadolinium. However, GBM and Single MET both have enhanced core and significant peri-tumor edema on the ceT1W images. These similar appearances on ceT1W images pose a challenge for preoperative GBM and MET differentiation. Therefore, developing effective computational methods to distinguish between these intracranial tumors is of great importance.

With advancements in computer vision and deep learning, various techniques for automated medical image recognition and analysis have made remarkable progress. From traditional machine learning algorithms to modern end-to-end deep neural networks (Chang et al., 2018), these innovations continue to enable intelligent classification and diagnosis of brain tumors based on MRI scans.

Some studies have applied machine learning and deep learning methods to analyze features of brain MRI images, achieving reasonable tumor classification and prediction accuracy. Blumenthal et al. utilized a support vector machine model to learn texture features from brain tumor images (Artzi et al., 2019), attaining moderate performance. Sohi et al. employed a deep convolutional neural network, obtaining 89.0% accuracy in tumor type prediction. However, most of these models rely on standard convolution operations, adapted from large-scale datasets like ImageNet, with little consideration of fine-grained, pixel-level semantic information (Tateishi et al., 2020). This limitation impedes learning of subtle, local lesion details.

To address this gap, we propose a novel deep learning model called SCAT-inception that optimizes module design based on the GoogLeNet architecture. SCAT-inception was tested and compared with other models in academic publications in this study.

## 2 Materials and methods

**Figure 1:**
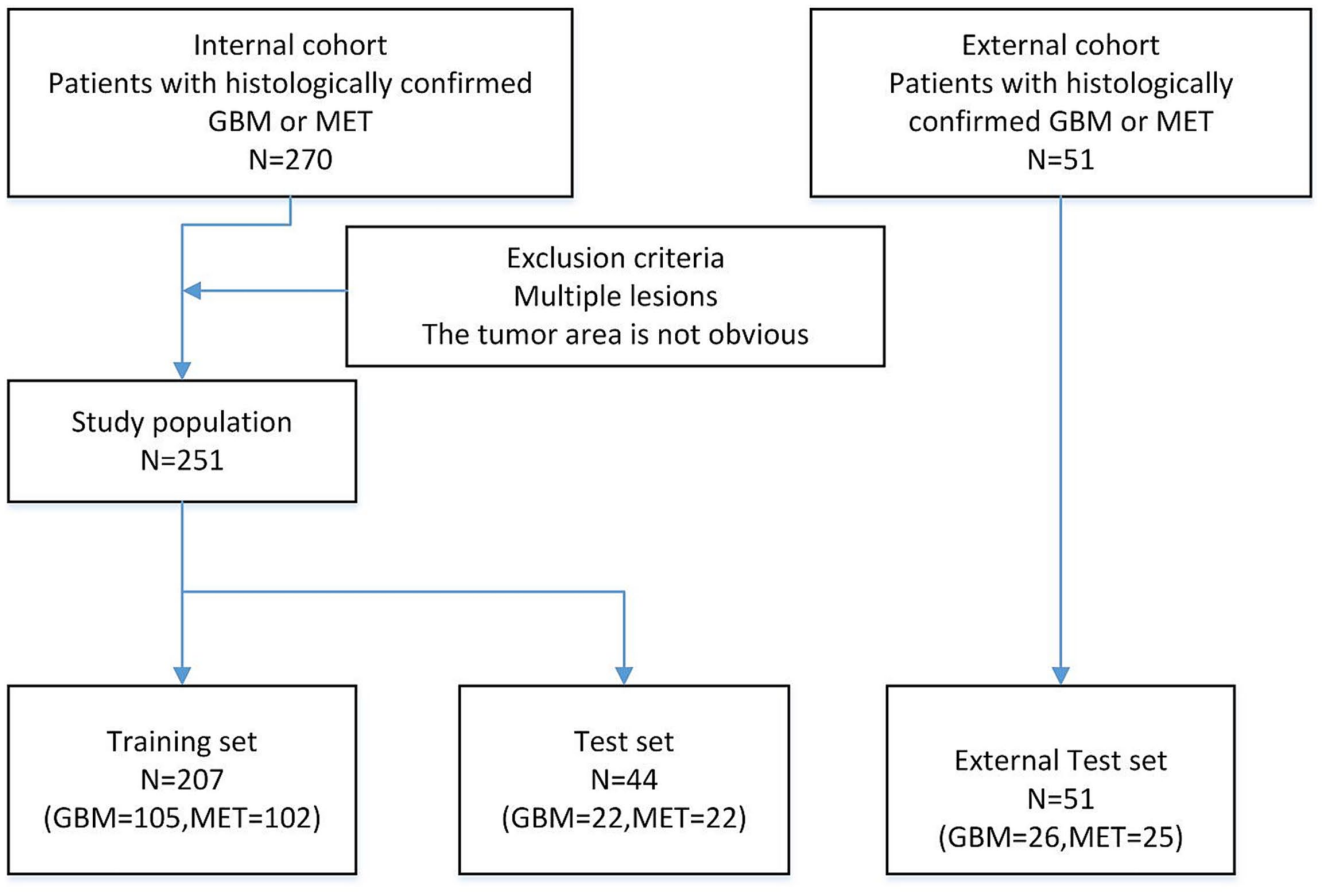
Flow chart showing the patient population.

As illustrated in Figure 1, the experiment collected axial enhanced T1 weighted (ceT1W) MRI images of 321 cases of high-grade gliomas and isolated brain metastases. 26 cases were randomly selected from the total number of GBM cases, and 25 cases were randomly selected from the total number of MET cases, for a total of 51 cases as the external test dataset. The remaining GBM and MET cases were merged, and 251 cases were selected from the 270 cases to compose the training dataset and test dataset. This study comprised 251 tumor patients from two clinical centers (Jinling Hospital and The Second People’s Hospital of Yibin), including 105 glioblastoma (GBM) cases and 102 brain metastasis (MET) cases. Using a five-fold cross-validation approach (Shin et al., 2021), 207 cases were partitioned into the training set, while the remaining 44 cases constituted the testing set. All included cases were solitary tumors, excluding those with prior surgical resection or multiple metastases. The patient age ranged from 35 to 70 years, with approximately equal gender distribution.

### 2.1 Experimental procedure

#### 2.1.1 Slices selection and ROI segmentation

**Figure 2:**
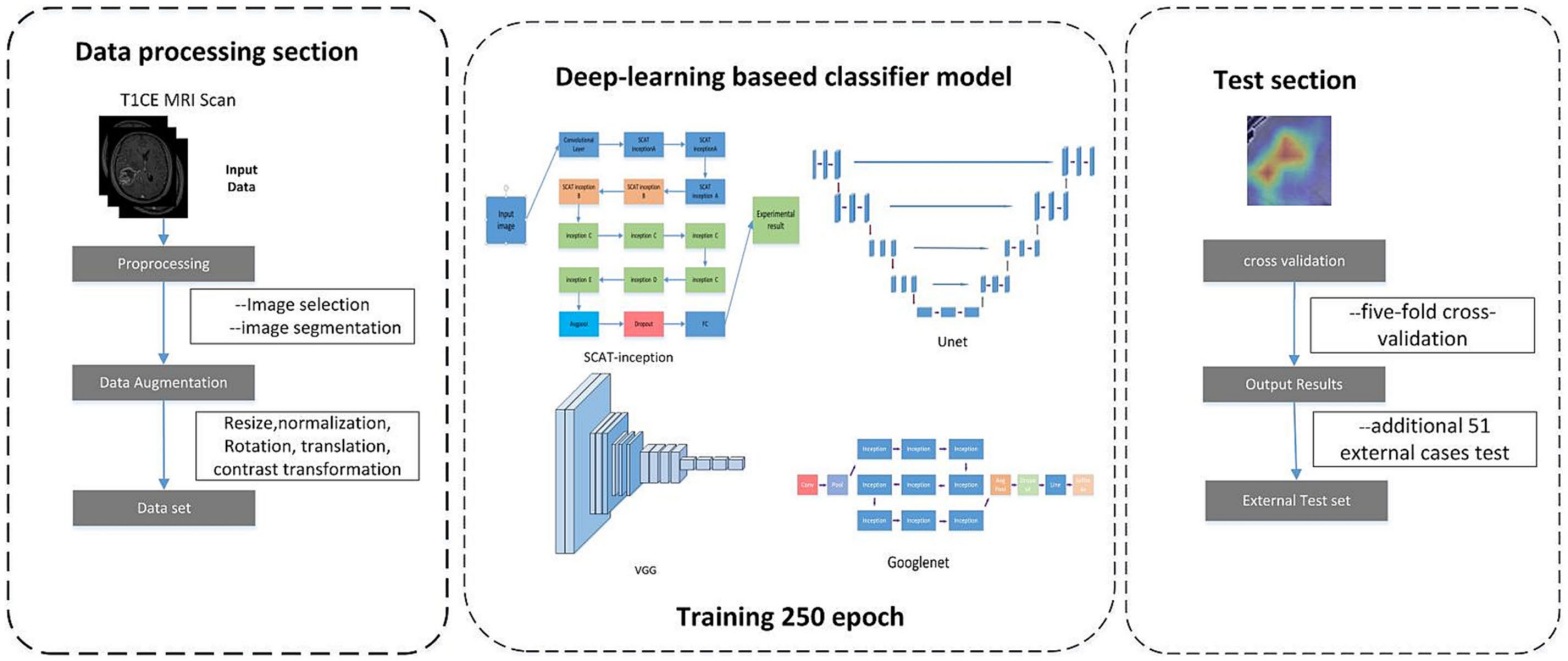
Flowchat of Deep learning develepment.

As shown in Figure 2, all the ceT1W images were collected from 3 MRI scanners of the two clinical centers. The 1.5-T scanner (Erlangen, Siemens Espree, Germany) was used to obtain MRI images of all patients before surgery. DICOM images of axial T1CE with a thickness of 1 mm were collected. The parameters for T1CE were as follows, Slicer thickness = 1 mm, Field-of-view = 130 mm, Flip angle = 15°, Echo time = 3.02 ms, Matrix size = 512 × 512 × 176, Repetition time = 1,650 ms, and Voxel dimensions = 0.997 × 0.997 × 1 mm3. The tumor regions were segmented via 3D Slicer software by two radiologists to extract the tumor core and surrounding edema, constructing three distinct datasets: core, edema, and overall. Considering the impact of dataset quality on model training, images with clearly delineated lesions were selected as candidate datasets. For each case, three representative slices exhibiting prominent pathological features were chosen, constituting a total analysis dataset of 753 slices. Of these, 621 slices across 207 cases were assigned as the training dataset, while the remaining 44 cases with 3 slices each formed the testing dataset.

#### 2.1.2 Data augmentation

Data augmentation was implemented on the constructed datasets, encompassing cropping of tumor sub-regions, pixel value range normalization, data centering, random rotation and translation, and color contrast transformation. Post-augmentation, the image corpus tripled to 1863 slices, which expands dataset size, enhances sample diversity, improves adaptation to varied features, and promotes generalization capability.

#### 2.1.3 Model training and validation

The training subsets were propagated through the SCAT-inception deep neural network for optimization. A five-fold cross-validation strategy was adopted for performance evaluation. Specifically, all 251 cases were partitioned into five distinct sets. The model was built on the PyTorch framework, leveraging the Adam optimizer with a learning rate of 1e-2 over 250 epochs. For each cross-validation fold, accuracy, sensitivity, and specificity were computed on the testing split. Upon completing five-fold validation, the mean and variance of these metrics were derived. Finally, an additional 51 external cases were utilized to evaluate generalization ability via accuracy calculation.

### 2.2 Network model architecture

Owing to the relative paucity of available medical imaging data, shallow network architectures may lack sufficient feature learning and fitting capacities. Conversely, deep networks with copious parameters risk overfitting. To surmount these challenges, we propose the SCAT-inception network, achieving balanced feature extraction and generalization through judicious depth and width enhancement. The SCAT-inception algorithm encompasses multiple Inception modules (Szegedy et al., 2015).

**Figure 3:**
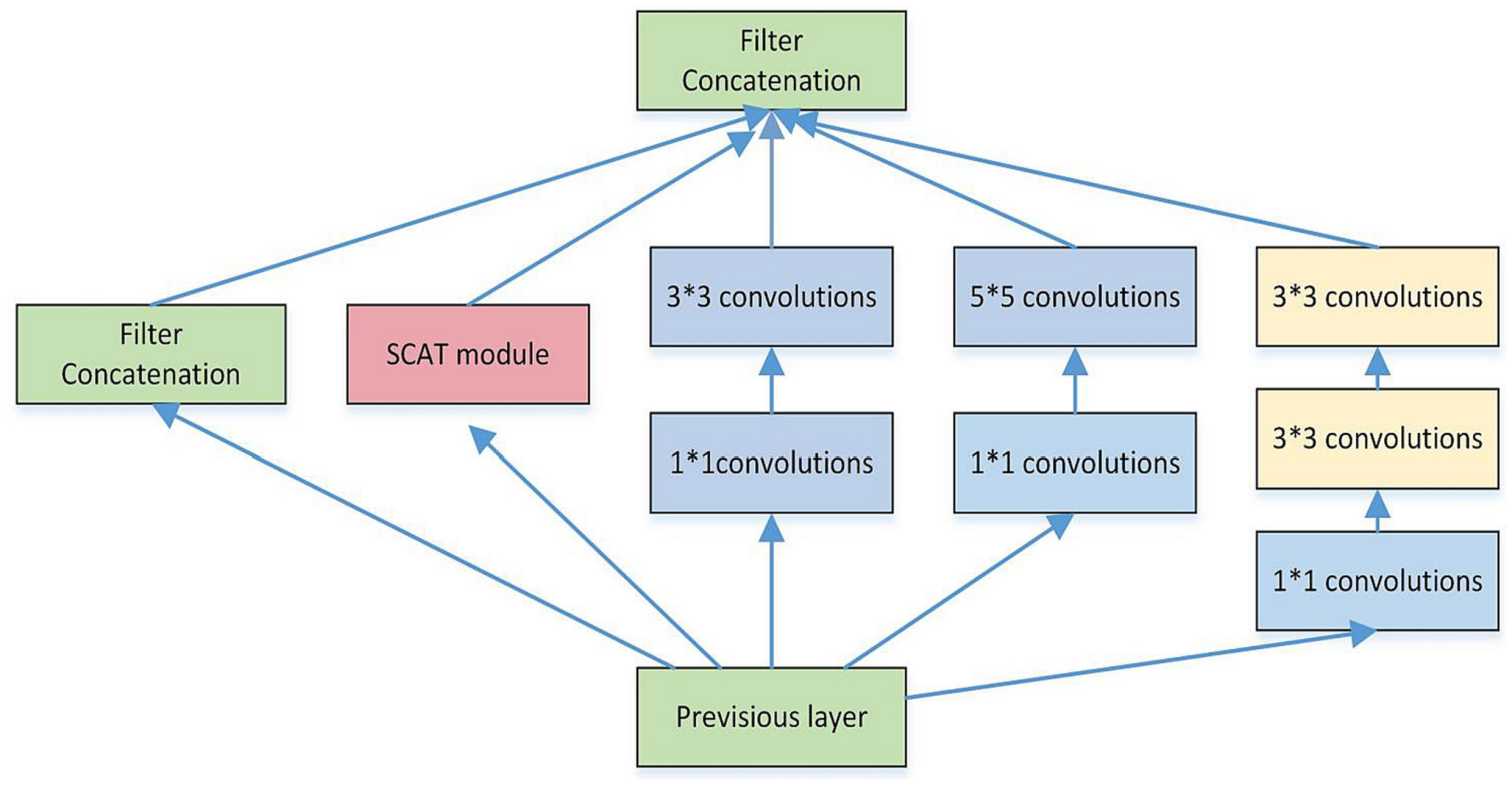
The structure of the SCAT inception.

**Figure 4:**
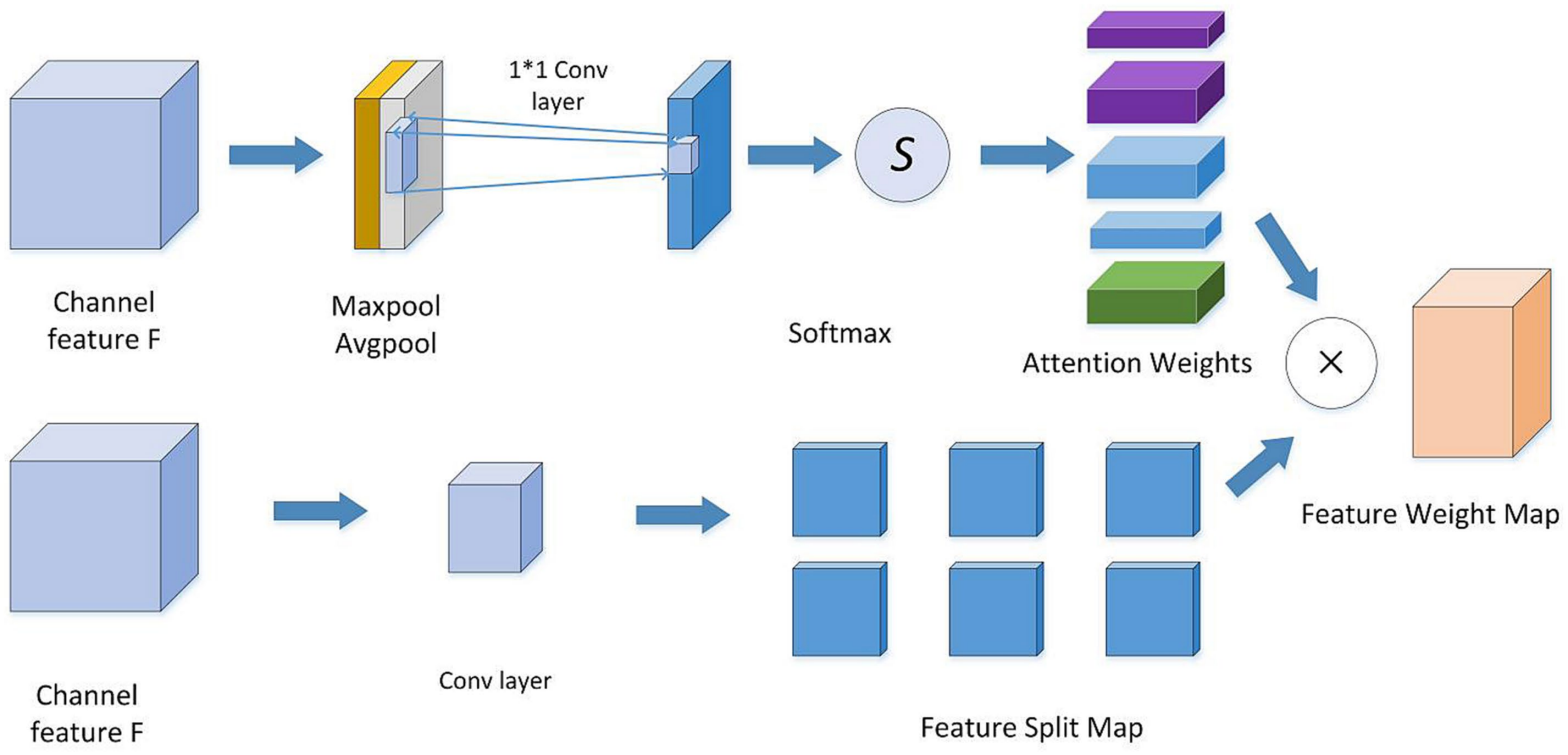
Spatial attention convolutional structure diagram.

As depicted in Figure 3, a spatial convolutional attention module termed SCAT is incorporated into the Inception structure. As depicted in Figure 4, this SCAT module first reduces the dimensionality of the feature maps using a 1 × 1 convolution, followed by another 1 × 1 convolution to generate channel-wise attention vectors. The attention vectors are subsequently softmax-normalized to derive attention weights, which are applied in an element-wise manner to the feature maps, enabling the network to learn pixel-level attention distributions.

Each SCAT-inception module encompasses convolutional blocks of assorted sizes, including 1 × 1, 1 × 3, 3 × 3, and 5 × 5 kernels (Szegedy et al., 2016). The width of the inception modules was set to 5, ensuring model stability. Different convolution operations can capture local image features from distinct perspectives (Skogen et al., 2019; Romano et al., 2022), thereby enabling collaborative target recognition. Compared to alternatives, the SCAT-inception architecture strikes an efficient balance between global and local feature learning. It combines the multi-branch design of Inception modules with varied receptive fields to concurrently learn global and local representations. By integrating pixel-level spatial attention modules like SCAT, it further acquires sensitivity to fine-grained details. This fusion of global context and localized attention allows comprehensive feature learning and expression. Relative to single-scale models, SCAT-inception demonstrates superior performance and efficiency.

**Figure 5:**
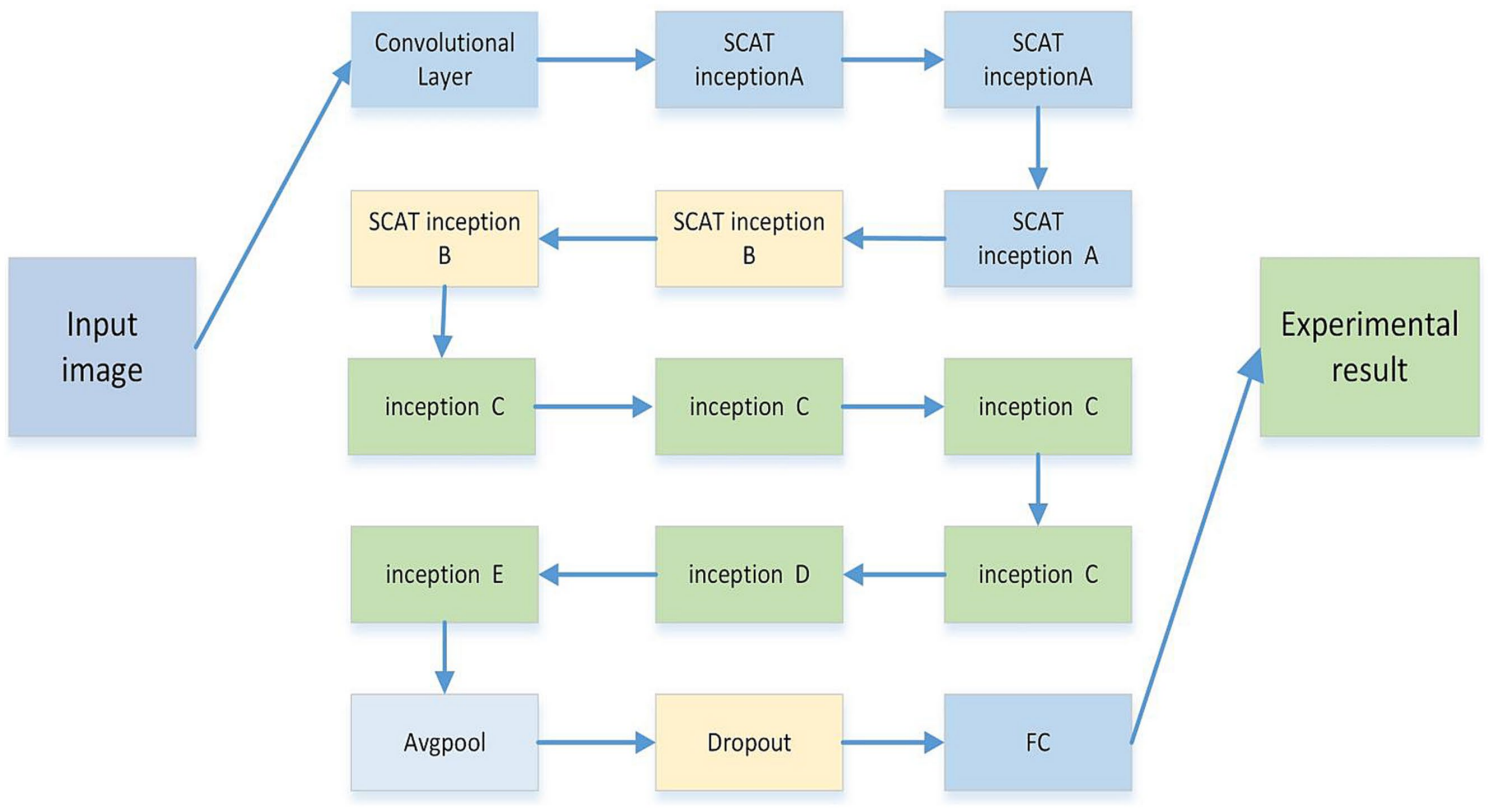
The model is composed of multiple improved inception structures of different sizes. Each inception structure is composed of 1 * 1, 3 * 3, 5 * 5, SCAT, etc. The whole model is composed of multiple inception modules, followed by average pooling, Dropout, and linear networks.

As shown in Figure 5, the SCAT-inception network comprises multiple convolutional layers and diverse Inception modules. Specifically, it contains 3 SCAT Inception-A units, 2 SCAT Inception-B units, 4 Inception-C units, 1 Inception-D unit, and 1 Inception-E unit, followed by global average pooling, Dropout, and fully-connected classification layers. The multi-scale convolutions within the Inception modules enable joint learning of global and localized image features (Sunwoo et al., 2016).

### 2.3 Model performance evaluation

In this study, T1-weighted contrast-enhanced (T1CE) preoperative MRI data were utilized, comprising a total of 251 glioblastoma and brain metastasis samples (Cha et al., 2007). Among these, 207 samples were assigned to the training set, while 44 samples constituted the test set.

The hyperparameters for model training were configured as follows: 250 epochs, a learning rate of 0.01, and a dropout rate of 0.5. The evaluation metrics employed in this study included accuracy, sensitivity, and specificity, calculated using the following Equations 1–3:


(1) $$\text{Accuracy}=\left(\text{TP}+\text{TN}\right)/\left(\text{TP}+\text{FN}+\text{TN}+\text{FP}\right)$$

(2) $$\text{Sensitivity}=\text{TP}/\left(\text{TP}+\text{FN}\right)$$

(3) $$\text{Specificity}=\text{TN}/\left(\text{TN}+\text{FP}\right)$$

In the classification task, glioblastoma (GBM) samples were designated as positive cases, while brain metastasis (MET) samples were denoted as negative cases. In the prediction outcomes, True Positives (TP) represent positive samples correctly classified by the model, True Negatives (TN) are negative samples correctly predicted, False Negatives (FN) denote negative samples incorrectly classified, and False Positives (FP) are positive samples incorrectly predicted.

### 2.4 Models training of VGG, UNet, and GoogLeNet

Several classic models including VGG, UNet (Cao et al., 2021), and GoogLeNet were trained using the same dataset, to serve as experimental comparisons. The hyperparameters for those model training were configured as follows: 250 epochs, a learning rate of 0.01, and a dropout rate of 0.5, the recognition results of several classic models are shown in Table 1.

**Table 1:** Accuracy of the three models for prediction.

Data	Model	Accuracy	Sensitivity	Specificity
T1CE	VGG	75.6%	76.7%	74.5%
T1CE	UNet	79.3%	81.2%	77.4%
T1CE	GoogLeNet	89.5%	91.3%	88.7%
T1CE	SCAT-inception	92.3%	93.5%	91.1%

Compared with models such as VGG, UNet, and GoogLeNet, the SCAT initial algorithm consists of different types of initial modules and includes spatial attention convolution modules. This enables the model to retain important information and recognize microscopic features in images such as brain tumors, while VGG is only composed of simple linear convolutional layers, UNet uses downsampling and upsampling structures, but it is more commonly used in the field of image segmentation. The GoogLeNet module is composed of identical repeating components, and its recognition feature direction is weaker than SCAT insertion. The diversity of SCAT feature extraction brings better performance than the unified structure of other networks lacking universal feature recognition.

## 3 Experimental results

Experiments were conducted via five-fold cross-validation on tumor core (Girshick et al., 2014), edema, and overall lesion images. The results are tabulated in Table 2. The accuracy on tumor core recognition reached 92.3%, while the edema and overall image accuracies were 85.5 and 87.8%, respectively. The core region performance exceeded that of edema and overall images, potentially attributable to more archetypal lesions with enhanced discriminative clues in the tumor core.

**Table 2:** Five-fold cross-validation accuracy table.

Class	Fold 1	Fold 2	Fold 3	Fold 4	Fold 5	Average
Core	92.3%	91.6%	93.4%	90.8	93.5%	92.3%
Overall parts	89.8%	88.7%	90.4%	87.5%	82.5%	87.8%
Edema	87.5%	85.4%	83.2%	86.7%	84.5%	85.5%

**Figure 6:**
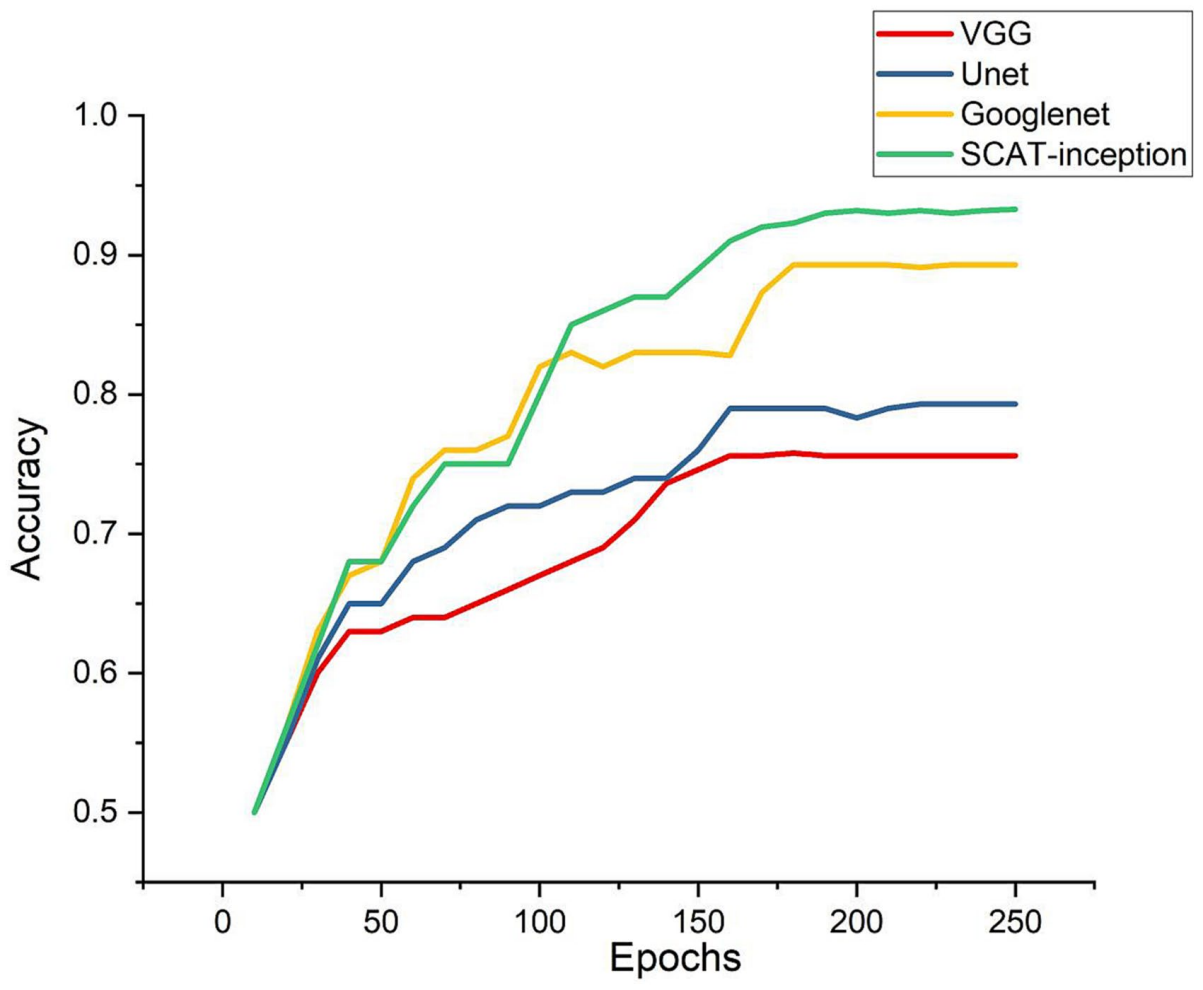
Accuracy of the four models during Model training process.

As depicted in Figure 6, test set accuracy exhibited continual improvement with more training epochs, eventually plateauing and validating effective model optimization. In this study, four models were compared: VGG, U-Net, GoogLeNet, and SCAT-inception. The accuracy, sensitivity, and specificity of each model are detailed in Table 1. The SCAT-inception model achieved 92.3% accuracy, 93.5% sensitivity, and 91.1% specificity. The VGG model attained 75.6% accuracy, 76.7% sensitivity, and 74.5% specificity. For the U-Net model, the metrics were 79.3% accuracy, 81.2% sensitivity, and 77.4% specificity. GoogLeNet yielded 89.5% accuracy, 91.3% sensitivity, and 88.7% specificity. Moreover, SCAT-inception produced 91.5% accuracy on external validation, demonstrating superior recognition performance compared to other models and validating the stability and efficacy of the proposed approach.

**Figure 7:**
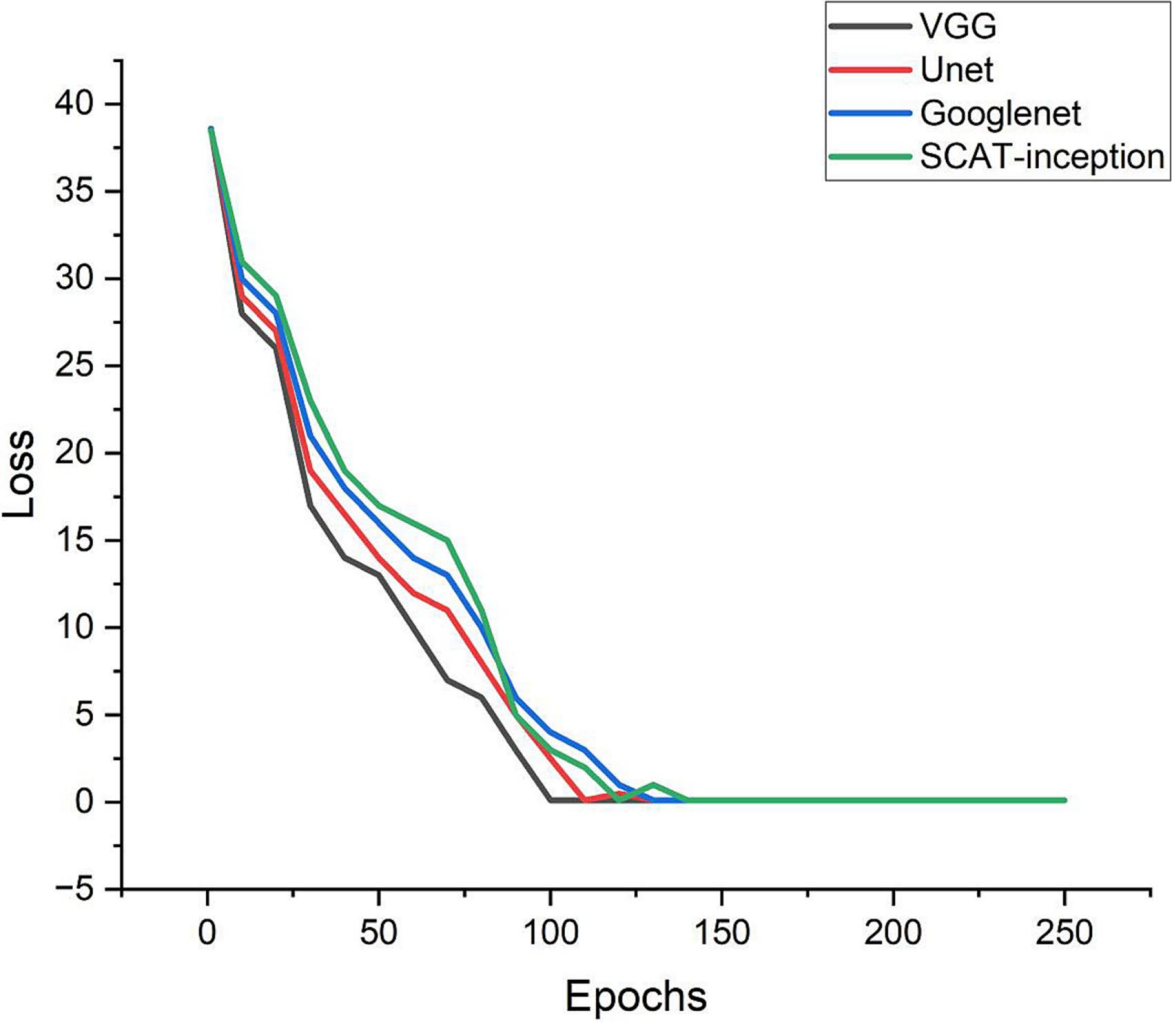
Loss curves of the four models during training process.

**Figure 8:**
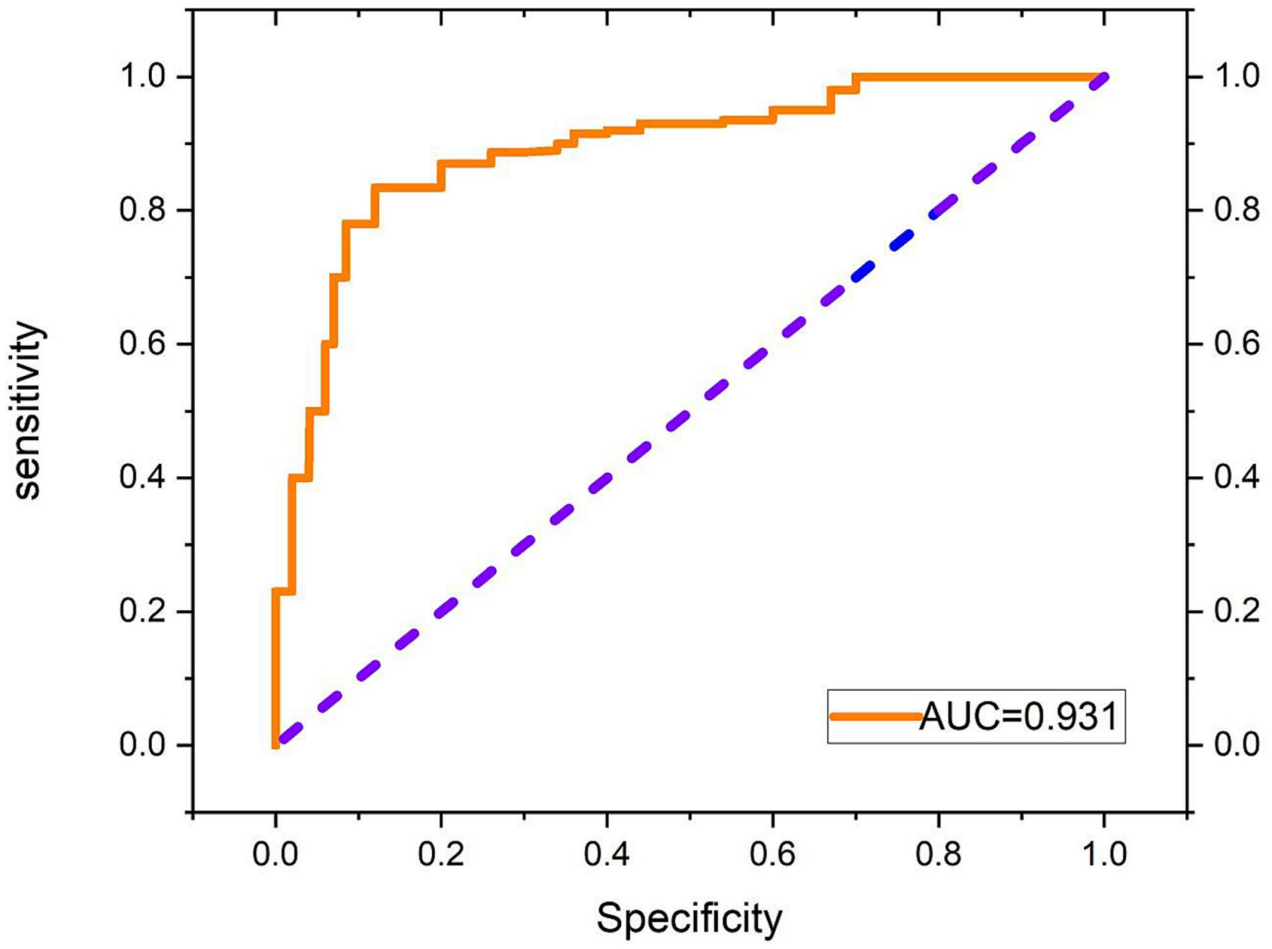
Receiver operating characteristic (ROC) curve of the classifier.

Additionally, the loss function of the SCAT-inception model exhibited a continuous decreasing trend (Figure 7). By the 100th epoch, the loss converged close to 0, indicating effective lesion feature learning by the model. Figure 8 shows the Receiver Operating Characteristic (ROC) curve of the classifier, with an AUC value of 0.931.

## 4 Conclusion

In this study, we propose a novel deep learning model called SCAT-inception for classifying glioblastoma and brain metastasis in MRI scans. By incorporating spatial convolutional attention within Inception modules, the method effectively emphasizes crucial local lesion patterns (Kamson et al., 2013; Takao et al., 2022). Experimental results demonstrate that SCAT-inception achieves 92.3% accuracy and 93.5% sensitivity, outperforming several classical models and attaining clinically viable performance.

In summary, the key innovations and contributions of this study are:

1. Integration of spatial convolutional attention modules into the Inception architecture to enable adaptive learning of lesion attention distributions.
2. Design of an efficient network balancing global and localized feature extraction.
3. Training and validation of robust recognition efficacy on a brain tumor MRI dataset.

This work provides an effective deep learning solution for computer-assisted diagnosis of brain tumors. Future directions involve validating generalization on large-scale multi-center data and exploring multimodal integration.

## 5 Discussion

The SCAT-inception network achieves effective differentiation of glioblastoma and brain metastasis in MRI by integrating spatial attention mechanisms into the Inception modules to emphasize crucial local lesion attributes (Amemiya et al., 2022; Zhang et al., 2023). The experimental findings demonstrate the robust classification capabilities of SCAT-inception. This proficiency can be attributed to several key architectural components: Adoption of multi-scale Inception modules to concurrently learn global and localized features. Patial attention dynamically adjusts feature map weight distributions along the spatial dimension, accentuating pathological details. The synergy between these two aspects facilitates extraction of complementary discriminative characteristics across levels. Additionally, the network underwent iterative optimization to ensure clinical viability.

Spatial attention convolution is a convolutional neural network architecture that incorporates spatial attention mechanisms into standard convolution operations to automatically learn the more important spatial locations in the input feature maps of the current task, thereby improving model performance. An attention layer is added after the standard convolution layer to generate a spatial attention map, where each value on the attention map indicates the importance of the features at the corresponding location. At the same time, the dot product is performed between the convolution layer’s attention map and output feature map to achieve the effect of adjusting feature responses according to spatial locations. The feature responses at important locations are amplified while unimportant locations are suppressed. Additionally, the attention map is generated by a simple convolutional subnetwork that can be trained end-to-end with the main network. The spatial attention convolution mechanism was originally used for processing 2D images, and is mainly applied to 2D image analysis tasks in the field of computer vision, such as image classification, object detection, semantic segmentation, etc. Therefore, by adjusting attention based on spatial locations, modeling of key spatial information can be enhanced to improve the model’s ability to recognize important features.

The spatial convolutional attention (SCAT) module confers several advantages: The convolution operation retains spatial information and captures pixel-wise attention mappings, making it well-suited for images with fine-grained characteristics (Yan et al., 2023). The convolutional parameters are relatively compact, conferring higher computational efficiency. Additionally, the local connectivity intrinsic to convolutional layers is superior for modeling local attention interdependencies. Moreover, the convolutional realization of attention is readily integrated into convolutional networks, facilitating embedding within Inception blocks.

Previous studies have investigated MRI-based brain tumor classification (Zhou et al., 2017). Machiko et al. applied machine learning to analyze MRI texture patterns, attaining 78% accuracy in distinguishing glioblastomas from metastases using 260 cases. Qian et al. extracted radiomic signatures from 412 MRI scans and tested various machine learning models, affirming the utility of radiomics for classification. Shin et al. developed a deep ResNet model, achieving over 88.9% accuracy on 598 samples. Relative to prior works, this study demonstrates competitive performance with fewer training cases of 321, enabled by the tailored SCAT-inception design. Future efforts could expand the multi-center case collection to augment the data pool. While only MRI modalities were assessed, incorporating multimodal cues could further boost performance. Overall, this study puts forth an efficacious deep learning solution for precise brain tumor discrimination that warrants continued optimization and investigation.

Although spatial attention convolution has shown effectiveness in emphasizing information regions, current methods also have some limitations. The fixed attention mode limits the adaptability to deformation, while the addition of models also increases computational costs. Moving forward, solutions include lightweight implementations, regularization techniques, and incorporation of dynamic attention concepts to simultaneously retain high performance and versatility. Integration with interpretability analysis methods would further bridge the gap between outstanding results and model transparency. Looking forward to the forefront of the development of the examination evaluation mechanism in the future, in order to unleash the full potential of applications in both visual and non visual fields.

## Data availability statement

The original contributions presented in the study are included in the article/supplementary material, further inquiries can be directed to the corresponding authors.

## Author contributions

CV: Data curation, Formal analysis, Investigation, Methodology, Project administration, Software, Validation, Writing – original draft, Writing – review & editing. X-JS: Data curation, Methodology, Resources, Writing – original draft. HC: Data curation, Methodology, Resources, Writing – review & editing. JQ: _. SP: Project administration, Validation, Writing – review & editing. KY: Project administration, Validation, Writing – review & editing. S-BC: Methodology, Project administration, Resources, Writing – review & editing. HR: Data curation, Methodology, Writing – review & editing.
